# Assessing PCR-Positive Acanthamoeba Keratitis—A Retrospective Chart Review

**DOI:** 10.3390/microorganisms12061214

**Published:** 2024-06-17

**Authors:** Frank Blaser, Anahita Bajka, Felix Grimm, Simone Metzler, Didier Herrmann, Daniel Barthelmes, Sandrine Anne Zweifel, Sadiq Said

**Affiliations:** 1 Department of Ophthalmology, University Hospital Zurich, University of Zurich, 8091 Zurich, Switzerlandsaidsadiq@gmx.ch (S.S.); 2 Institute of Parasitology, University of Zurich, 8057 Zurich, Switzerland; 3 Institute of Optometry, University of Applied Science, 4600 Olten, Switzerland

**Keywords:** Acanthamoeba keratitis, infectious keratitis, contact lens wear, diagnostic challenge, co-infections, polymerase chain reaction

## Abstract

Ophthalmologists’ diagnostic and treatment competence in Acanthamoeba keratitis varies widely. This investigator-initiated, retrospective, single-center chart review examined the electronic patient files regarding PCR-positive Acanthamoeba keratitis. We included corneal and contact lens assessments. We further reviewed the patient’s medical history, corneal scraping results regarding viral or fungal co-infections, and the duration from symptom onset to final diagnosis. We identified 59 eyes of 52 patients from February 2010 to February 2023, with 31 of 52 (59.6%) being female patients. The median (IQR, range) patient age was 33 (25.3 to 45.5 [13 to 90]) years, and the mean (SD, range) time to diagnosis after symptom onset was 18 (10.5 to 35 [3 to 70]) days. Overall, 7 of 52 (7.7%) patients displayed a bilateral Acanthamoeba infection, and 48 (92.3%) used contact lenses at symptom onset. Regarding other microbiological co-infections, we found virologic PCR testing in 45 of 52 (86.5%) patients, with 3 (6.7%) positive corneal scrapings. Fungal cultures were performed in 49 of 52 (94.2%) patients, with 5 (10.2%) positive corneal scrapings. The medical treatment success rate was 45/46 (97.8%). This study raises awareness of patient education in contact lens handling and screens for further microbial co-infections in suspected Acanthamoeba cases.

## 1. Introduction

Non-trachomatous corneal opacities are the fifth leading cause of blindness worldwide, with infectious keratitis being the most prevalent condition [1,2]. The incidence and causative agents of infectious keratitis vary depending on geographical, environmental, and patient-specific risk factors, including contact lens use, ocular trauma, immune status, and hygiene practices [3]. Among the causative agents, *Acanthamoeba* spp., ubiquitous, free-living single-celled organisms globally found in soil, dust, and water resources, are particularly noteworthy. Acanthamoeba can tolerate harsh environmental conditions such as pH or temperature fluctuations. Hence, classification criteria based on immunological or physiological behavior cannot successfully identify the various different Acanthamoeba species due to their variable organism profiles under different growth conditions [4]. Currently, the different species are identified using morphological criteria as well as DNA sequencing [5]. The corneal infection due to Acanthamoeba is mainly attributed to contact lenses, which are increasingly used in the developed world [6]. In financially disadvantaged countries, the main association represents ocular trauma [7]. Because Acanthamoeba keratitis accounts for only up to 9 cases per 100,000 corneal infections [8], its clinical presentation is often subacute or chronic, and it may mimic other corneal pathologies; misdiagnosis with resulting unfavorable visual outcomes is common [6,9]. In fact, half of the cases of vision loss due to microbial keratitis are caused by Acanthamoeba [10].

After corneal epithelial adhesion by protein-binding mechanisms, the infectious organisms destroy the epithelium and invade the Bowman membrane through different cytopathic effects such as cytolysis, phagocytosis, or inducing apoptosis [5,11]. Once within the corneal stroma, Acanthamoeba generate different proteases to induce the host collagenolytic effect [12,13], resulting in the characteristic ring infiltrate seen on slit lamp examination. Further, in vitro examinations have found a chemotactic affinity of Acanthamoeba to corneal nerves [14], which may explain the perineural infiltrates and the severe pain out of proportion to the clinical signs in infectious states. Interestingly, florid Acanthamoeba keratitis seems to stabilize in its progression and rarely leads to infectious endophthalmitis. Even though in vitro studies have demonstrated cytolytic effects on endothelial cells, which in theory allows the entrance into the anterior chamber, there are only few case reports of Acanthamoeba endophthalmitis [15,16].

The clinical presentation of Acanthamoeba keratitis may resemble other, more common infectious causes. Hence, the final diagnosis is based on detecting the mentioned organism. Traditionally, culture assessment using non-nutrient *Escherichia coli* agar plates and different stains such as Giemsa or calcofluor white has been the most widely accepted method of diagnosis [17]. However, the plates must be re-evaluated daily for at least a week and are labor-intensive [17]. Hence, polymerase chain reaction (PCR) and imaging are often adjunctively used in vivo confocal microscopy, enabling a significantly faster time to diagnosis of within an hour. Real-time PCR carries an excellent specificity of 100% and a sensitivity of 94% in detecting Acanthamoeba, which poses the question of whether to adopt this method as the new gold standard [18]. In vivo confocal microscopy examines the infected cornea for round, hyperreflective objects with a double-ring appearance, representing Acanthamoeba cysts [19]. Therefore, this method enables both an immediate visualization and an evaluation of the treatment response [19].

Our tertiary care facility in Switzerland serves as a national referral center for various atypical keratitis cases, including suspected Acanthamoeba infections. Referring ophthalmologists’ diagnostic and treatment competence in Acanthamoeba keratitis varies widely. However, timely diagnosis and appropriate management significantly impact visual outcomes [20]. In light of these challenges, this retrospective study aims to examine the patient and clinical characteristics of PCR-confirmed cases of Acanthamoeba keratitis at our institution and to review the current diagnostic and therapeutic regimens.

## 2. Materials and Methods

This is an investigator-initiated, retrospective, single-center study conducted at the University Hospital Zurich in Switzerland. We identified patients diagnosed with PCR-positive Acanthamoeba keratitis in our tertiary care facility between February 2010 and February 2023. The leading ethics committee in Zurich waived our study protocol (BASEC number 2023-01146). We handled all data according to Good Clinical Practice guidelines.

### 2.1. Data Collection

We reviewed our electronic medical files of patients aged 18 and older regarding PCR-positive Acanthamoeba results, including corneal scrapings and contact lens examinations. We assessed the identified patients’ medical histories regarding contact lens use. Further, we investigated for herpes simplex virus (HSV)-1, HSV-2, varicella–zoster virus (VZV), and fungal co-infections, again considering virologic PCR results and fungal cultures for corneal scrapings and contact lens assessments. Due to the presumably high prevalence of bacterial co-infections, the high rate of culture positivity in corneal scrapings due to probe contamination, and the fact that we treat all infectious keratitis patients with topical antibiotics in our clinic before a definitive diagnosis, we did not test for bacterial co-infections in this study. However, all corneal scrapings routinely include bacterial investigations. Moreover, we evaluated treatment success independent of functional outcome and defined it as the number of patients not requiring surgical intervention such as therapeutic penetrating keratoplasty. We considered surgical intervention as treatment failure, as it represents the more definite, invasive therapeutic option. Finally, we evaluated the findings of anterior-segment optical coherence tomography obtained using Heidelberg Spectralis version 1.9.10.0 and in vivo confocal microscopy using Heidelberg Spectralis HRT 3 RCM version 1.5.2.0 (both by Heidelberg Engineering GmbH, Heidelberg, Germany).

### 2.2. Acanthamoeba Detection

Sample preparation and DNA extraction: Liquid samples containing corneal scrapings were centrifuged (3 min, 14,000× *g*), and the ‘pellet’ (although invisible in most cases), together with 200 microliters (μL) of the supernatant, was used for DNA isolation in 1.5 milliliter (mL) Eppendorf tubes. If samples contained a ‘corneal brush’, the brush was cut into two small pieces and added to the fluid. Contact lens care or storage solution samples of 2 ml were centrifuged and treated as above. Contact lenses were cut in half in disposable sterile petri dishes using sterile scalpel blades. One half was further cut into about five small pieces. Pieces were transferred to a 1.5 mL Eppendorf tube for DNA isolation. The second half of the lens served as a backup sample. DNA was isolated from all sample types using the tissue protocol of the QIAamp DNA mini kit (Qiagen, Hilden, Germany) according to the manufacturer’s instructions. After the proteinase K digestion step, samples containing solid particles were centrifuged (1 min, 14,000× *g*), and the supernatant was used for the following steps. A sample of 5 µL of the final DNA eluate (DNA concentrations of 6.0–22.4 ng/µL, estimated using a NanoDrop One spectrophotometer, Thermo Fisher Scientific, Zurich, Switzerland) was used per reaction.

DNA amplification: The primers and TaqMan probe used to amplify part (105 bp) of the 18S rRNA gene were Aca-F1 (5’-CCC AGA TCG TTT ACC GTG A-3’) [21], Aca-R1 (5’-GAG GAC AGG GTC CTA TTC CA-3’), and Aca-S1 (5’-FAM-TTC TGC CAC CGA ATA CAT TAG CAT GGG ATA-BHQ1-3’), all synthesized by Microsynth (Microsynth AG, Balgach, Switzerland). Primers were analyzed using the online OligoAnalyzer tool (Integrated DNA Technologies, Coralville, IA, USA). A risk of self- or hetero-dimer formation was not identified. Samples were tested in duplicate in a reaction volume of 25 μL. TaqMan Fast Advanced Master Mix (applied biosystems by Thermo Fisher Scientific, Zurich, Switzerland) was used with 900 nM of each primer and 200 nM of probe. In silico analysis showed that acanthamoeba genotypes T1 to T15 were recognized. This included genotype T4, which is most frequently found in Acanthamoeba keratitis, as well as the rarer keratitis genotypes T2, T3, T5, T6, T8, T9, T11, T13, and T15 [22]. Serial dilution experiments using *Acanthamoeba castellanii*, strain 2HH (genotype T4), grown in vitro showed that positive reactions can be expected with a DNA amount equivalent to 1–5 cells per reaction. No reaction with genomic DNA of Balamuhia, Naegleria (both kindly provided by N. Müller, Bern, Switzerland), Hartmanella (kindly provided by J. Walochnik, Vienna, Austria), Enterocytozoon, Encephalitozoon, Entamoeba, Toxoplasma, Leishmania, Plasmodium Trypanosoma, or Babesia was observed. Amplifications were run in a 7900HT Fast Real-Time PCR System (Applied Biosystems by Thermo Fisher Scientific, Zurich, Switzerland) under standard conditions (2 min at 50 °C, 10 min at 95 °C and 45 cycles of 15 sec at 95 °C and 1 min at 60 °C). All samples were tested in duplicate. If both reactions gave positive signals at fewer than 38 cycles, samples were judged as positive. Weak positivity means that the positive signals were detected between 38 and 42 cycles. Positive and negative (no template) controls were included in all runs. The absence of potential inhibitory effects and the amplification efficiency were monitored by adding 5 μL DNA eluate to an additional reaction amplifying a part of the 18S rRNA gene of *Phytophthora citricola*, a plant pathogen.

### 2.3. Treatment Regimen

**Figure 1:**
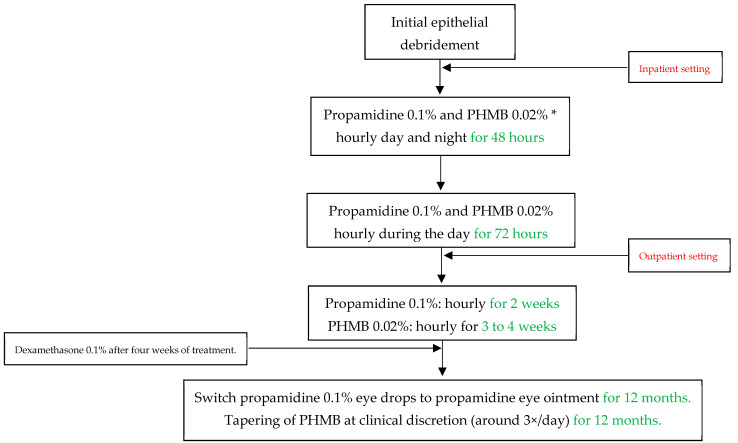
Acanthamoeba keratitis treatment protocol at the University Hospital Zurich in Switzerland. PHMB = Polyhexamethylene biguanide. * Chlorhexidine 0.02% can be used equivalent to and in the same dose as PHMB if the latter is not available.

All patients underwent conservative treatment according to our standard of care at the University Hospital Zurich, as displayed in Figure 1. The antiamoebic treatment included initial epithelial debridement to reduce pathogen density and to improve drug penetration. In an inpatient setting, we first applied propamidine 0.1% and polyhexamethylene biguanide (PHMB) 0.02% eye drops hourly for 48 h (during the day and at night), and then reduced the frequency to hourly during the awake time for another 72 h. After five days and in an outpatient setting, we further reduced propamidine to every other hour for two weeks and PHMB to every other hour for three to four weeks, depending on the severity of the infectious keratitis and the treatment toxicity. We then switched the propamidine eye drops to propamidine ointment, which we advised the subjects to apply at night. We tapered PHMB at clinical discretion, and it was mostly used three times per day for twelve months. We regarded chlorhexidine 0.02% eye drops as equivalent to PHMB, which we used in the same regimen in case PHMB was not readily available. However, this case never occurred, so that no patients had to be treated with chlorhexidine. We produced propamidine in the cantonal pharmacy in Zurich as a magistralis formulation, and only if this was otherwise not possible did we resort to the commercial product Brolene^®^ (0.1% propamidine isethionate, Thornton & Ross, West Yorkshire, UK). After four weeks of treatment, we introduced dexamethasone 0.1% eye drops to account for immunogenic reactions during the treatment. The dosage of dexamethasone was based on clinical judgement, and we stopped the corticosteroid treatment at least one month before discontinuing antiamoebic therapy. During the treatment period, we paid particular attention to initiating adequate pain relief with systemic non-steroidal anti-inflammatory drugs or opioids if necessary, as well as administering cycloplegic eye drops if this was helpful.

### 2.4. Statistical Analyses

We applied descriptive statistics, presenting means with standard deviation and medians with interquartile ranges (IQR), or minimum to maximum values for continuous data and numbers and percentages for categorical data.

## 3. Results

**Figure 2:**
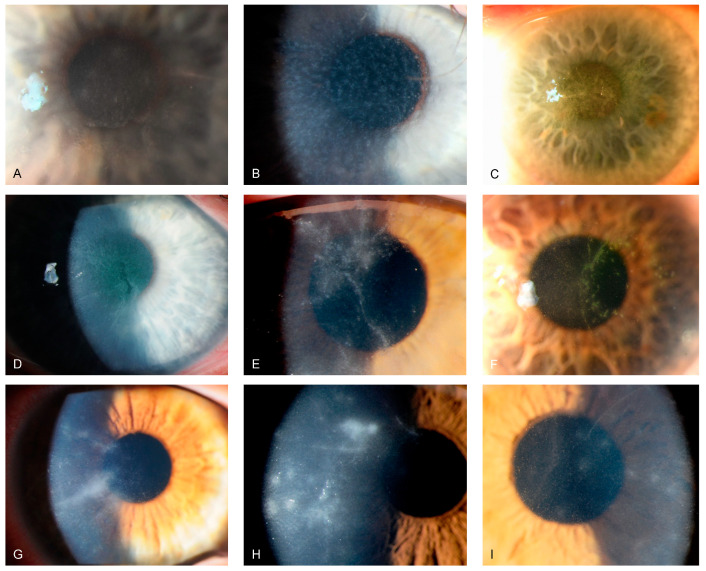
Slit-lamp photographs of different patients with Acanthamoeba keratitis, obtained by direct illumination or scleral scatter. (**A**–**C**) show greyish epithelial opacifications and microcysts (“dirty epithelium”). (**D**–**F**) display pseudo-dendritiformic epitheliopathy. (**G**–**I**) depict perineural infiltrates (“radial keratoneuritis”).

**Figure 3:**
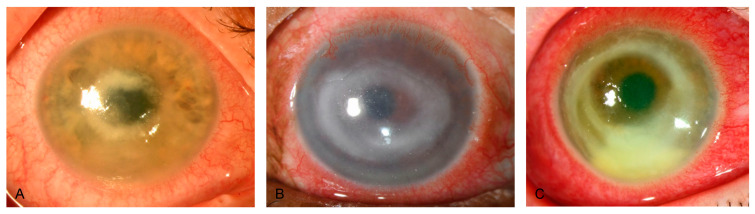
Slit-lamp photographs of different patients with Acanthamoeba keratitis, obtained by direct illumination. (**A**–**C**) show deep stromal ring infiltrates, a late sign of Acanthamoeba corneal infection.

Reviewing our medical files from February 2010 to February 2023 regarding PCR-positive Acanthamoeba keratitis, we identified 59 eyes of 52 patients, with 31 of 52 (59.6%) being female patients. The maximum number of annual cases was 10 in 2022. Across the investigated period, we did not observe an increasing trend of PCR-positive Acanthamoeba keratitis at our study site. Of the 52 patients, we found 9 (17.3%) infections during spring, 18 (34.6%) during summer, 16 (30.8%) during autumn, and 9 (17.3%) during winter. The median (IQR, range) patient age was 33 (25.3 to 45.5 [13 to 90]) years, and the mean (SD, range) time to diagnosis after symptom onset was 18 (10.5 to 35 [3 to 70]) days. Overall, 7 of 52 patients (7.7%) displayed bilateral Acanthamoeba keratitis, and 48 (92.3%) used contact lenses at symptom onset. Table 1 depicts patient demographics and characteristics, and Table 2 further provides contact lens information. Of 46 (2.2%) treated patients, 1 required emergent penetrating keratoplasty due to conservative treatment failure, resulting in a conservative treatment success rate of 97.8%. Figure 2 and Figure 3 show slit-lamp photographs of patients with findings of Acanthamoeba keratitis.

We performed corneal scrapings in all patients, whereby 39 of 52 (75%) were PCR-positive for Acanthamoeba. We examined contact lenses in 21 of 52 (40.4%) patients, and 20 of 21 (95.2%) returned positive. One patient displayed a negative contact lens result, whereby we detected Acanthamoeba in the corneal assessment. Table 3 shows the PCR evaluation in more detail. Regarding other microbiological co-infections, we found virologic PCR testing in 45 of 52 (86.5%) patients. Of these, 2 (4.4%) were positive for HSV-1, none (0%) were positive for HSV-2, and 1 (2.2%) was positive for VZV, resulting in a total of 3 of 45 (6.7%) viral co-infections. Further, we found fungal cultures in 49 of 52 (94.2%) patients. Of these, we detected positive fungal cultures in 5 of 49 (10.2%) corneal scrapings and in 6 of 21 (28.5%) contact lens assessments.

Regarding the antiamoebic therapy, the median (IQR [range]) time to treatment in days was 18 (10.5 to 35 [3 to 70]). All but 1 of the 39 (97.4%) positive corneal scrapings were treated; the mentioned patient displayed weak positivity in PCR testing and an unfitting clinical presentation. For the contact lens investigations, 13 of 20 (65%) patients were treated against Acanthamoeba. Table 3 displays the correlation between PCR results and treatment.

## 4. Discussion

This study retrospectively investigated our local database regarding PCR-confirmed Acanthamoeba keratitis cases. Over thirteen years, we identified 52 patients, most being young adults. The diagnosis was often delayed by a median of 18 days after symptom onset. Most patients used contact lenses, although the proportion of rigid contact lens wearers appeared to be overrepresented. Viral or fungal co-infection seemed considerable. The overall medical treatment success rate was surprisingly high. With Acanthamoeba keratitis being a potentially sight-threatening corneal infection [23], increased awareness among ophthalmologists remains crucial.

The number of reported cases has increased dramatically since Jones et al. first described a patient with Acanthamoeba keratitis in 1973 [24]. Our study cohort had a median age of 33 years and a more or less balanced gender distribution, in line with the reported literature [23,25,26]. Young adults may be more prone to infectious risk behaviors such as wearing contact lenses. In addition, we detected most infections during summer or autumn, when recreational activities such as swimming, bathing, and traveling are more common. Despite a continuous rise in the average annual temperature in Switzerland [27], we did not record an increasing trend in infections over the observation period. Acanthamoeba displays two different stages in its life cycle, where the cyst stage represents a dormant phase and the trophozoite stage a vegetative, possibly infectious one [4]. Excystation occurs with optimal conditions, and the temperature plays an important role [28]. In vitro, on agar plates, the optimal excystation temperature is 30 degrees Celsius [28], which may explain a higher rate of infections during the warmer season or in warmer climates [29].

Similar to findings from the literature [8,30,31], we also found a delayed time to diagnosis at our clinic. Based on clinical findings, Acanthamoeba keratitis can be classified into early, advanced, and late stages. The early clinical signs may begin within the first two weeks of infection and mainly include epithelial changes such as punctate epithelial lesions, microcystic changes, or pesudodendritiformic epitheliopathy, which are not specific to Acanthamoeba and may lead to misdiagnosis of other, more common types of microbial keratitis [32,33,34]. Although not pathognomonic, as it also occurs in Pseudomonas keratitis, infiltration along the corneal nerves (known as radial keratoneuritis) may be one of the most seminal clinical signs in the early stages of infection [35]. A protracted clinical course may lead the patient to develop severe sequelae, such as deep stromal ring infiltrates or corneal melting, often occurring within the first month of the disease and requiring more invasive treatment options such as therapeutic keratoplasty [34]. Late signs may follow months after the primary infection and include sequelae such as scleritis, sterile anterior uveitis with subsequent anterior synechiae, secondary glaucoma, atrophic iris changes, cataracts, and rarely retinal vasculitis, and chorioretinitis [34]. When Acanthamoeba keratitis is suspected, ophthalmologists should have a low threshold for referring patients to medical facilities with appropriate diagnostic tools, such as in vivo confocal microscopy, PCR availability, or increased clinical expertise, to ensure timely management, ultimately improving visual prognosis.

This study found a conservative treatment success rate of 97.8%, which seems surprisingly high. The two most extensive case series reported medical cure rates of around 61% (138 of 227 patients) when mainly applying topical PHMB [36], and around 71% (158 of 224 patients) with mainly topical chlorhexidine [37]. Dart et al. investigated PHMB at a higher concentration of 0.08% as monotherapy and found it to be non-inferior to the dual therapy of the commonly used 0.02% PHMB plus propamidine [38], which is still lower than our reported cure rate. Historically, treatment for acanthamoeba keratitis has focused on early penetrating keratoplasty, as antimicrobial success rates were poor. While the trophozoites are amenable to antimicrobial drugs, the dormant cysts are highly resilient. Currently, the leading conservative treatment options include biguanides, aromatic diamidines, antibiotics, and steroids [6]. The biguanides PHMB and chlorhexidine are active against both amoeba forms as they enter the pathogen and increase their cytoplasmatic membrane permeability, resulting in cell destruction and death [39]. Nevertheless, biguanides may show considerable corneal epithelial toxicity and elevate the intraocular pressure due to excessive inflammation [39]. The mode of action of the aromatic diamidines propamidine and hexamidine involves the inhibition of DNA and RNA synthesis, which allows amoebicidal and cysticidal effects to occur [40]. Neomycin is the most commonly prescribed antibiotic in this context. However, neomycin is not cysticidal, which limits its applicability as a standalone treatment against acanthamoeba [6]. Instead, neomycin is often used in combination with biguanides or aromatic diamidines [6]. Finally, steroids remain controversial because, although they reduce the concomitant inflammatory stromal reaction, they may increase the amoebae pathogenicity by promoting excystation and thus increasing the trophozoite load [41]. Moreover, early steroid use may improve the initial clinical picture and thus mask the true extent of the infectious keratitis, which could lead to a delayed diagnosis. We believe that our conservative treatment success rates are exceptionally high due to our strict therapy protocol, as we treat all patients in an inpatient setting once the diagnosis is established or if there is a high clinical suspicion. This method allows for a high degree of treatment adherence with an intensive protocol (hourly day and night for the first two days), which is crucial in the early infection phase. In addition, we keep all outpatient follow-up visits at our clinic and do not discharge the patients to aftercare with ophthalmologists in private practice, who have less therapeutic expertise. Hence, due to the relatively high conservative success rate, we are sticking to the treatment regime at our clinic despite current therapeutic developments, such as higher-dose PHMB as monotherapy. Nevertheless, there is still a great need for an effective drug with lower side effects, which would entail a simpler treatment regime with fewer patient consultations.

Most of our investigated patients used contact lenses at the time of symptom onset, which is certainly a risk factor for corneal infection. Regarding the lens type, soft contact lenses are a well-reported risk factor for acanthamoeba keratitis [39], while reports associated with rigid contact lenses are limited [42]. The increased comfort of soft lenses may cause patients to engage in riskier behavior, such as showering, swimming, or sleeping with them, which is more prevalent among patients who only wear contact lenses occasionally (e.g., once a week) and have less experience with proper hygienic care [39]. In Switzerland, as in most European countries, soft contact lenses are the most used [43]. According to expert opinions and research by the Swiss Opticians Association, the estimated relative distribution of rigid contact lenses is around 8% of all lens types in Switzerland [44]. Our study found that 12.5% of the patients with PCR-positive Acanthamoeba keratitis used rigid contact lenses at symptom onset, which is not only a high number, but also an overrepresentation of the mentioned lens type. Our data show that advising patients to use rigid contact lenses to reduce contact lens-associated infections is unjustified. We believe that proper education on how to minimize the risk behaviors associated with lens wear and care habits is more valuable than low-evidence advice on lens type.

The literature discusses exciting relationships between the co-existence of fungi and Acanthamoeba. One hypothesis assumes an endosymbiosis of certain fungi with Acanthamoeba, which enables the fungi to multiply within the encapsulated amoeba, allowing protection from macrophage degradation [45]. In addition, certain fungi, such as Fusarium, may use endosymbiosis not only to stimulate their growth, but also to promote Acanthamoeba excystation [46]. This synergistic trait may facilitate and enhance co-pathogenicity. Still, the most common misdiagnosis for Acanthamoeba keratitis is herpetic infection [37], where topical steroids would unfavorably be initiated. Identifying co-infections is of utmost clinical relevance to improve visual outcomes and reduce the need for surgical intervention. If financially feasible, we encourage PCR testing for viral co-infections and fungal cultures in suspected Acanthamoeba cases.

The strengths of this study include the long duration of the retrospective review and the considerable number of patients included. However, this study has several limitations. First, the retrospective design is inherently prone to selection bias and missing data. Within the timeframe of this study, our clinic changed our standard operating procedure, whereby we now routinely assess for bacterial, virologic, fungal, and Acanthamoeba infection in all corneal scrapings for infectious keratitis and assess patients with high suspicion routinely using in vivo confocal microscopy. Before, we only investigated for clinical suspicion. The common rate of co-infections and misdiagnoses led us to adapt our clinical practice. Second, the overall sample size is comparably moderate. The true prevalence of investigated data, such as the rate of co-infections or percentage of contact lens users, may differ. Third, this study was performed in a tertiary care center in central Europe. Patient demographics may differ elsewhere in the world.

## 5. Conclusions

In summary, most patients with Acanthamoeba keratitis used contact lenses at the time of symptom onset. In our patient cohort, rigid contact lenses were overrepresented compared to Switzerland’s population distribution of lens types. This is why the dogma of higher safety from microbial keratitis, at least regarding acanthamoeba infection, seems unjustified. The delayed time to diagnosis and a considerable amount of viral or fungal co-infections raises awareness for a lower threshold to refer patients to appropriate clinical centers for suspected amoeba infections and to screen for further microbial co-infections. Finally, the strict treatment protocol in this study has shown a comparatively high cure rate, which confirms its success in tackling this resilient pathogen. Even though we will maintain this protocol in the near future, the need for newer and effective treatment modalities is still unmet.

## Figures and Tables

**Table 1:** Patient demographics and characteristics. IQR = interquartile range, OD = right eye, OS = left eye.

Time Period of Chart Review	February 2010 to February 2023
Total patients; *N*	52
Total eyes; *n*	59
Sex; female	31 (59.6%)
Age median (IQR [range]); years	33 (25.3–45.5 [13 to 90])
Unilateral; *n*	45 of 52 (86.5%)
OD	26 of 52 (50%)
OS	19 of 52 (36.5%)
Bilateral; *n*	7 of 52 (13.5%)
Time to treatment median (IQR [range]); days	18 (10.5–35 [3 to 70])

**Table 2:** Contact lens information, depicted as numbers and percentages.

Patients Using Contact Lenses; *N*	48 of 52 (92.3%)
Contact lens type	
Soft lenses	40 of 48 (83.3%)
Rigid lenses	6 of 48 (12.5%)
Scleral lenses	1 of 48 (2.1%)
Unclassified	1 of 48 (2.1%)
Soft contact lens use	
Daily use	6 of 40 (15%)
Twice-weekly use	9 of 40 (22.5%)
Monthly use	22 of 40 (55%)
Cosmetic lenses	1 of 40 (2.5%)
Unclassified	2 of 40 (5%)

**Table 3:** Acanthamoeba polymerase chain reaction (PCR)-positive results and numbers with percentages of patients who received antiamoebic treatment. All patients either had PCR-positive results in corneal scrapings, contact lens assessments, or both.

	PCR Assessments	Treated Patients
Positive corneal scrapings	39 of 52 (75%)	38 of 39 (97.4%)
Negative corneal scrapings	13 of 52 (25%)	6 of 13 (46.2%)
Positive contact lens testing	20 of 21 (95.2%)	13 of 20 (65%)
+ positive corneal scrapings	7 of 21 (33.3%)	7 of 7 (100%)
+ negative corneal scrapings	13 of 21 (61.9%)	6 of 13 (46.2%)
Negative contact lens testing	1 of 21 (4.8%)	1 of 1 (100%)

## Data Availability

Data will be made available upon request to the corresponding author (due to ethical reasons).
